# *Phellodendron amurense* Rupr. Polysaccharides protects against diabetic nephropathy via alteration of PI3K/GSK-3β/Nrf2/TGF-β/Smad signaling pathway and gut microbiota

**DOI:** 10.1007/s13659-025-00566-z

**Published:** 2026-01-10

**Authors:** Mei Mei, Huawei Sun, Kai Zhang, Feng Zhang, Shiqing Sun, Yu Zhang

**Affiliations:** 1 School of Basic Medicine, Jiamusi University, Jiamusi, 154007 China; 2 The First Affiliated Hospital, Jiamusi University, Jiamusi, 154002 China; 3 College of Pharmacy, Jiamusi University, Jiamusi, 154007 China

**Keywords:** *Phellodendron amurense* Rupr. Polysaccharides, Diabetic nephropathy, Gut microbiota

## Abstract

In this study, a diabetic nephropathy (DN) rat model was established using 2% Streptozocin (STZ) solution, and an in vitro DN model was constructed by stimulating HK-2 cells with 30 mM glucose to investigate the mechanism of *Phellodendron amurense* Rupr. Polysaccharides (PAP) in ameliorating DN. Results demonstrated that PAP, a neutral homogeneous polysaccharide with molecular weight of 1.98 × 105 Da composed of Rha, GalA, Gal, and D-Xyl, exerted renal protective effects through multiple pathways. It enhanced renal antioxidant capacity and alleviated oxidative damage in DN by upregulating PI3K/AKT pathway-related protein expression. Simultaneously, PAP activated the TGF-β/Smad pathway via Nrf2 to mitigate renal fibrosis symptoms in DN, while inhibiting cellular apoptosis. Furthermore, PAP suppressed renal inflammation through gut microbiota reduction, thereby protecting against renal injury in DN rats. This study reveals that PAP alleviates DN symptoms by modulating gut microbiota, enhancing antioxidant and anti-fibrotic capacities, and inhibiting apoptotic pathways, comprehensively elucidating its multifaceted therapeutic mechanisms against DN.

**Graphical Abstract:**

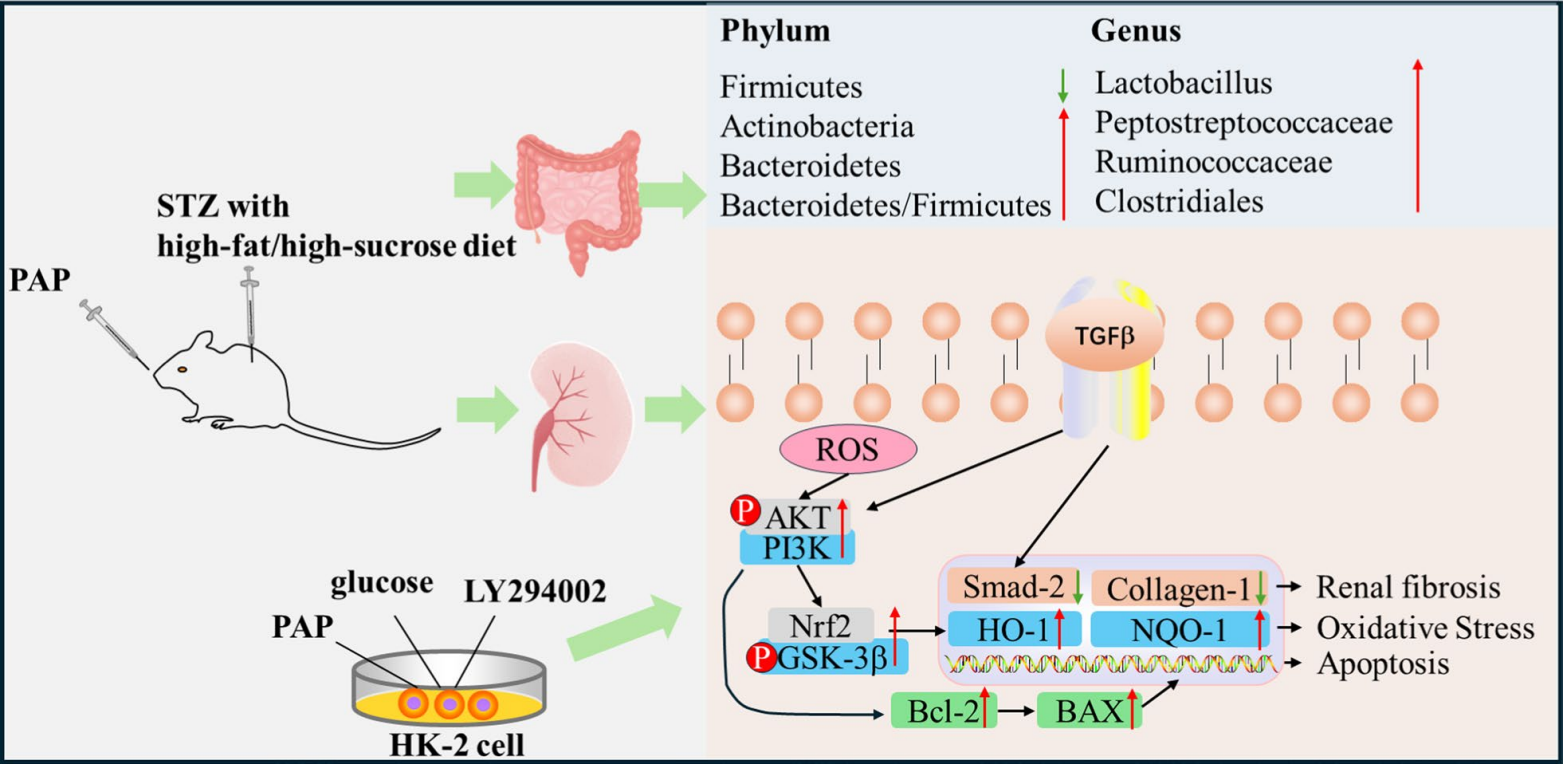

## Introduction

Diabetic nephropathy (DN) is a metabolic disorder characterized by structural renal damage and abnormal renal function induced by diabetes mellitus. In its advanced stages, DN progresses to end-stage renal disease (ESRD), ultimately resulting in death. Distinct from other diabetes-related complications, DN specifically involves glomerular hyperfiltration, podocyte injury, renal tubular dysfunction, and extracellular matrix accumulation, culminating in renal fibrosis and glomerulosclerosis [1]. According to the 2023 Diabetic Kidney Disease Report released by the International Diabetes Federation (IDF) highlights that 30–40% of global diabetic patients will ultimately develop DN [27]. Heretofore, DN management primarily focuses on correcting glucose metabolism disorders and hemodynamic abnormalities in diabetic patients. Furthermore, due to the rising prevalence of DN, it is anticipated that diabetic patients will develop chronic kidney disease requiring renal replacement therapies (such as dialysis or transplantation) [11, 24]. These therapeutic approaches impose substantial economic and psychological burdens while consuming extensive medical resources [19]. Therefore, promoting therapeutic strategies for DN amelioration significant clinical importance.

DN is a complication induced by diabetes mellitus, occurring when elevated blood glucose levels lead to renal injury. The progression of metabolic processes underlying this disease is complex, encompassing various factors such as insulin resistance, oxidative stress, renal fibrosis, and apoptosis [5]. In diabetic patients, impaired antioxidant defense mechanisms may be activated, while diabetes-induced high levels of advanced glycation end products (AGEs) promote reactive oxygen species (ROS) production, resulting in oxidative stress and cellular structural damage. Several pro-inflammatory and pro-fibrotic signaling pathways are activated, including TGF-β/Smad, Wnt/β-catenin, and PI3K/Akt, all of which contribute to the progression of DN [22, 30]. When insulin binds to its receptor (IR) on the cell surface, it initiates downstream signaling through tyrosine kinase activity, activating pathways including the PI3K/Akt signaling pathway and MAPK/ERK signaling pathway [7]. Dysregulation of the PI3K/Akt pathway can lead to hyperactivation of mTOR signaling, causing glomerular hypertrophy, podocyte injury, and renal fibrosis, while inducing ROS production that further exacerbates renal damage [25]. Additionally, renal fibrosis in DN arises from TGF-β responsiveness to hyperglycemia and AGEs/ROS-induced oxidative stress. Following activation of AGEs/ROS, PKCβ, and CTGF, TGF-β binds to TGF-β R1 and TGF-β R2 [20], triggering downstream signaling that activates receptor complexes and recruits phosphorylated SMAD2 and SMAD3 proteins, ultimately influencing fibrosis-related processes. Therefore, suppressing ROS generation to alleviate oxidative stress, renal fibrotic responses, and apoptosis represents a critical strategy for mitigating DN [26].

Natural products have long been explored as medicinal resources for treating various major diseases. Substantial experimental evidence from both preclinical studies and clinical practice has demonstrated the potential efficacy of natural products in alleviating renal injury. In preclinical studies, numerous natural products have recently been reported to mitigate diabetic nephropathy through modulation of oxidative stress, inflammation, and apoptosis. In recent years, the role of gut microbiota in regulating diabetic nephropathy has attracted growing research attention. As a natural product, polysaccharides are widely distributed in most plants. Cyclocarya paliurus polysaccharides alleviate type II diabetes symptoms by modulating gut microbiota and short-chain fatty acids [26]. Corn silk polysaccharides ameliorate diabetic nephropathy through restoration of intestinal microecology [6]. Various tea polysaccharides exhibit anti-diabetic activity via antioxidant mechanisms [12]. Collectively, these studies indicate that polysaccharides possess regulatory potential against diabetes and its associated complications.

Studies have demonstrated that alkaloids from *Phellodendron amurense* Rupr. exhibit potential in treating diabetes mellitus and its related complications [17]. The aqueous extract of *Phellodendron amurense* Rupr. alleviates diabetic symptoms in rats by reducing lipid peroxidation [14], while its neutral polysaccharide component displays anti-diabetic activity and concurrently inhibits osteoporosis in diabetic rats [33]. In this study, PAP and their structures were investigated. We aim to determine whether *Phellodendron amurense* Rupr. polysaccharides (PAP) can ameliorate diabetic nephropathy symptoms and explore the underlying mechanisms by which PAP alleviates diabetic nephropathy.

## Materials and methods

### Materials

*Phellodendron amurense* Rupr. from Huanan County (Jiamusi, China). All monosaccharide standards were obtained from Shanghai yuanye Bio-Technology Co., Ltd (shanghai, China). HE staining kit, Masson staining kit, PAS staining kit and TUNEL staining kit were purchased from Meilunbio (Dalian, China). All Biochemica kits (FBG, urinary protein, Scr, and BUN) were provided by Nanjing Jiancheng Bioenginering Institute. (Nanjing, China). Antibodies against HO-1, NQO1, Nrf2, p-AKT, p-GSK3B, GSK3B, α-SMA, collagen1, p-SMAD, SAMD, TFG-β, Bax and Bcl-2 were purchased from Beyotime Biotechnology Co., Ltd. (Wuhan, China). All the other chemicals used were provided by Aladdin Chemical Reagent Co., Ltd. (Shanghai, China) and Beyotime Biotechnology Co., Ltd. (Wuhan, China).

### Preparation of *Phellodendron amurense* Rupr. Polysaccharides and determination of chemical composition

The pulverized herbal material was defatted with petroleum ether. The dried powder underwent reflux extraction twice with distilled water (2.5 h per cycle) at 100 °C. The resulting filtrates were pooled and concentrated under reduced pressure. Ethanol was introduced into the concentrated solution to a final concentration of 80% (v/v), and the mixture was allowed to stand overnight to facilitate precipitation. The precipitate was isolated by centrifugation. Free proteins in the polysaccharide-rich solution were eliminated using the trichloroacetic acid (TCA) method, followed by decolorization and further protein removal via AB-8 macroporous resin chromatography. The purified solution was concentrated and lyophilized to yield PAP and the yield was 6.32%.

The lyophilized PAP was analyzed for molecular weight using high-performance gel permeation chromatography (HPGPC). Monosaccharide composition of PAP was determined by high-performance liquid chromatography (HPLC). For Fourier-transform infrared (FT-IR, Thermo Scientific, USA) spectroscopy, 2 mg of PAP was mixed with 200 mg of potassium bromide (KBr), compressed into a pellet using a hydraulic press, and scanned on an FT-IR spectrometer within the wavenumber range of 4000–400 cm^−1^. PAP water solution after TCA (10 µ g/ml) was scanned by UV detector (UV, Agilent, USA). Morphological and structural characterization of PAP was performed via scanning electron microscopy (SEM, S-4800; Hitachi, Japan): 1 mg of PAP was mounted, and images were captured at magnifications of 50×, 100×, 200×, and 500×.

### Animals and experimental design

60 specific pathogen-free (SPF) male Sprague–Dawley (SD) rats (6–8 weeks old, 200 ± 20 g body weight) were provided by the Animal Experiment Center of Jiamusi University. Animals were housed under controlled conditions at 25 ± 2 °C with 50 ± 5% relative humidity and a 12/12-h light/dark cycle. All experimental procedures involving animals were conducted in accordance with the guidelines approved by the Institutional Animal Care and Use Committee of Jiamusi University.

After one week of acclimatization, the rats were randomly divided into six groups (n = 10/group): Control group, DN group (STZ 50 mg/kg), MET group (MET 100 mg/kg), PAP-low concentration (PAP-L) group (PAP 200 mg/kg), PAP- medium concentration (PAP-M) group (PAP 400 mg/kg), and PAP- high concentration (PAP-H) group (PAP 800 mg/kg). Excluding the control group, all other groups received a 2% STZ solution prepared in 0.1 mol/L citrate-sodium citrate buffer (pH 4.5, 4 °C). Prior to STZ administration, all rats were fasted for 10 h. The modeling groups were intraperitoneally injected with STZ, followed by free access to food and water. After 72 h, fasting blood glucose (FBG) was measured. Rats exhibiting FBG ≥ 16.7 mmol/L for three consecutive days were considered diabetic and subsequently fed a high-fat/high-sucrose diet. DN model was confirmed at week 4 by sustained hyperglycemia and urinary protein levels ≥ 30 mg. Except the control group and DN group, all diabetes nephropathy rats were assigned to orally the prescribed concentration of MET or PAP once a day for 8 weeks. The control group and DN group were given an equal amount of physiological saline orally. Blood glucose levels were monitored consistently throughout the experiment. At the end of 8 weeks, all rats were fasted for 12 h and anesthetized via intraperitoneal injection of 1% sodium pentobarbital (0.17 mL/100 g body weight). Fecal and blood samples were collected, followed by euthanasia via cervical dislocation. Kidney tissues were fixed in 4% paraformaldehyde, while other organs were flash-frozen in liquid nitrogen and stored at −80 °C for subsequent analysis.

### Cell culture

Human renal tubular epithelial cells (HK-2 cells) were cultured in Dulbecco’s Modified Eagle Medium (DMEM) supplemented with 10% fetal bovine serum (FBS). HK-2 cells induced with 5.5 mM glucose for 24 h served as the normal glucose control group, while those treated with 30 mM glucose for 24 h were designated as the high glucose group (HG). For the LY294002 treatment group, the high glucose-conditioned cells were further supplemented with 20 μM LY294002 (PI3K inhibitor). In the polysaccharide + LY294002 co-treatment group, cells were exposed to both 20 μM LY294002 and 100 μg/mL PAP under high glucose conditions. Following 24 h of continuous culture under the specified treatments, total proteins were extracted from each group for subsequent analysis.

### TUNEL staining

The HK-2 cells were analyzed using the In Situ Cell Death Fluorescein Detection Kit via TUNEL assay. Images were captured under an optical microscope. The number of TUNEL-positive cells was quantified in each group of images. The percentage of positive cells was determined using ImageJ software.

### Biochemical analysis

Fasting blood glucose (FBG) levels in different groups were measured at designated time points using a microplate reader. Serum levels of FBG, 24h urinary protein, serum creatinine (Scr), and blood urea nitrogen (BUN) were determined using commercial assay kits according to manufacturers’ protocols. Renal tissues were collected and processed to evaluate superoxide dismutase (SOD) activity, malondialdehyde (MDA) content, and glutathione (GSH) levels using specific detection kits (Nanjing Jiancheng Bioengineering Institute, China).

### Histological analysis

For pathological staining of renal tissues, the specimens were fixed in 4% paraformaldehyde, dehydrated, embedded in paraffin, sectioned, and subsequently stained with HE, MASSON, and PAS according to the specification.

### Western blotting

Kidney tissues or cultured HK-2 cells were retrieved from storage at −80 °C. For tissue samples, an appropriate amount (approximately 50–100 mg) was placed in a pre-chilled homogenizer. For cell samples, cells were washed twice with ice-cold PBS before collection. Samples were lysed in RIPA lysis buffer supplemented with 1 mM PMSF, a protease inhibitor cocktail, and a phosphatase inhibitor cocktail (Roche, Basel, Switzerland). Tissue samples were thoroughly homogenized on ice, while cell samples were lysed on ice for 30 min with intermittent vortexing to ensure complete lysis. The lysates were then centrifuged at 12,500 rpm for 15 min at 4 °C, and the supernatants containing total protein were carefully collected. Protein concentrations were determined using a BCA Protein Assay Kit. Equal amounts of protein (30 μg per lane) were mixed with 5× SDS-PAGE loading buffer and denatured by boiling at 100 °C for 10 min. Subsequently, the protein samples were separated by electrophoresis on 10% or 12% SDS–polyacrylamide gels and transferred onto polyvinylidene difluoride (PVDF) membranes using a wet transfer system. After transfer, the membranes were blocked with 5% bovine serum albumin (BSA) in Tris-buffered saline with 0.1% Tween-20 for 2 h at room temperature to block non-specific binding sites. The membranes were then incubated overnight at 4 °C with the following primary antibodies: rabbit anti-PI3K (1:1000,), rabbit anti-p-AKT (Ser473) (1:1000), rabbit anti-AKT (1:1000), rabbit anti-p-GSK-3β (Ser9) (1:1000), rabbit anti-GSK-3β (1:1000), rabbit anti-Nrf2 (1:1000), rabbit anti-HO-1 (1:1000), rabbit anti-NQO1 (1:1000), rabbit anti-TGF-β (1:1000), rabbit anti-α-SMA (1:1000), rabbit anti-Collagen I (1:1000), rabbit anti-Bcl-2 (1:1000), rabbit anti-Bax (1:1000), and mouse anti-GAPDH (1:10,000) as a loading control. Following overnight incubation, the membranes were washed three times with TBST for 10 min each. Subsequently, the membranes were incubated with horseradish peroxidase (HRP)-conjugated goat anti-rabbit or goat anti-mouse secondary antibodies (1:10,000) for 1.5 h at room temperature. After washing again with TBST for three 10-min intervals, the protein bands were visualized using an Enhanced Chemiluminescence (ECL) Detection Kit. Images were captured with a ChemiDoc Imaging System. Finally, the densitometry of the protein bands was quantified using ImageJ software. The relative expression of target proteins was normalized to that of GAPDH. Specifically, the levels of phosphorylated proteins (p-AKT, p-GSK-3β) were normalized to their corresponding total protein levels (total AKT, total GSK-3β).

### 16S rRNA gene sequencing

After euthanizing the rats, the cecal contents of rats were collected into 5 mL EP tubes and immediately preserved in ice boxes. Following sample collection, all EP tubes were flash-frozen in liquid nitrogen and subsequently shipped to Wuhan Frasergen Bioinformatics Co., Limited. PCR amplification sequencing targeting the V3-V4 regions was performed using specific primers (F: 5′-ACTCCTACGGGAGGCAGCA-3′; R: 5′-GGACTACHVGGGTWTCTAAT-3′), followed by Illumina platform-based amplicon sequencing of murine intestinal content microbiota. The obtained data were annotated against the Greengenes 13.8 database. Subsequent analyses included Alpha diversity, Beta diversity, and taxonomic composition assessments.

### Data analysis

The 16S rRNA sequencing data were analyzed and visualized using R language. Statistical analysis and graphical representation were performed with GraphPad Prism 9.0. Intergroup comparisons were analyzed by One-Way ANOVA, with *p* < 0.05 considered statistically significant. Experimental data are presented as mean ± SD.Pearson correlation analysis was used to determine the correlation between genus level of 16s rRNA and WB results in rats.

## Result

**Fig. 1:**
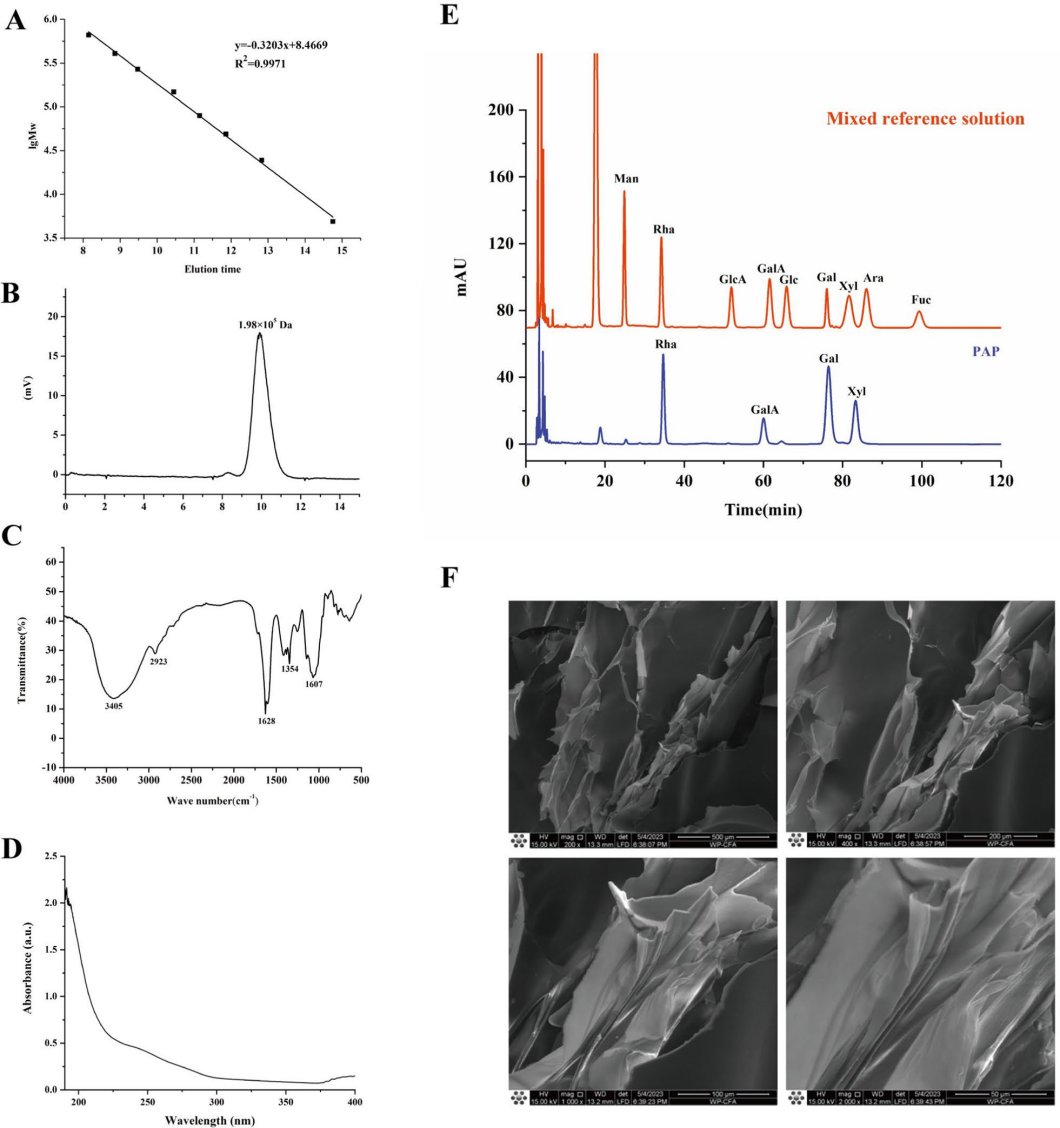
Chemical analysis of PAP. **A** standard curve of Molecular weight. **B** HPGPC chromatogram. **C** FT-IR spectrum. **D** UV image after protein removal by TCA. **E** monosaccharide composition. **F** SEM observation of PAP 50×, 100×, 200×, 500×)

The molecular weight distribution results indicated that PAP primarily exhibited a molecular weight of 1.98 × 10^5^ Da (Fig. 1A, B). The FT-IR spectrum of PAP (Fig. 1C) showed a strong absorption peak at 3405 cm^−1^, corresponding to the O–H stretching vibration in polysaccharides. A weak absorption peak at 2923 cm^−1^ was attributed to the C–H stretching vibration of methylene groups. Additionally, the absorption peak at 1628 cm^−1^ corresponded to C=O stretching vibrations, and the peak at 1607 cm^−1^ indicated the presence of uronic acid. UV full-scan spectra (190 nm~400 nm) show that the absence of significant absorption peaks at 280 nm confirms the absence of proteins in PAP (Fig. 1D). The monosaccharide composition of PAP consisted of rhamnose (Rha), galacturonic acid (GalA), galactose (Gal), and D-xylose (Xyl) (Fig. 1E). SEM (Fig. 1F) demonstrated that PAP formed ‌amorphous aggregates with irregular surface topography, a morphology typical of hygroscopic polysaccharides.

### PAP ameliorated renal injury in DN rats

**Fig. 2:**
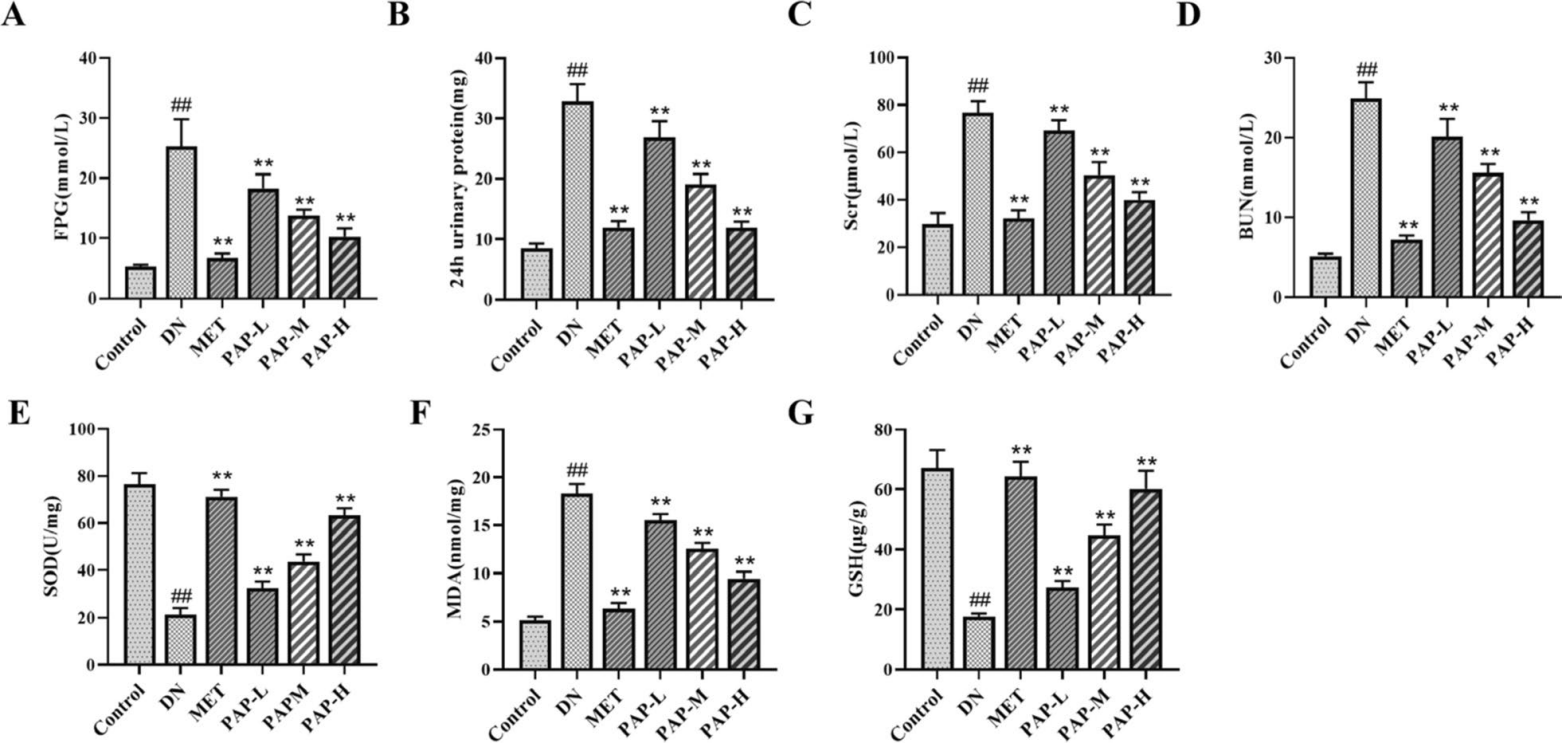
PAP treatment improved the renal function of the DN rats. Data were depicted as mean ± SD (n = 3). **A** The change of serum FBG. **B** The change of 24 h- urinary protein. **C** The change of serum Scr. **D** The change of serum BUN. **E** The change of renal SOD. **F** The change of renal MDA. **G** The change of renal GSH). ^##^*p* < 0.01vs. Control group; *p < 0.05, **p < 0.01 vs. the DN group

The levels of FBG, 24-h urinary protein, Scr, BUN, SOD, MDA, and GSH in renal tissues were measured. The results demonstrated that the DN group exhibited significantly higher levels of FBG, 24-h urinary protein, Scr, BUN, and renal MDA compared to the control group, while SOD and GSH levels were markedly lower than those in the control group (Fig. 2A–G). However, after 8 weeks of treatment with PAP, the levels of these biomarkers were significantly reduced or elevated compared to the DN group.

### Effect of PAP on kidney pathological injury

**Fig. 3:**
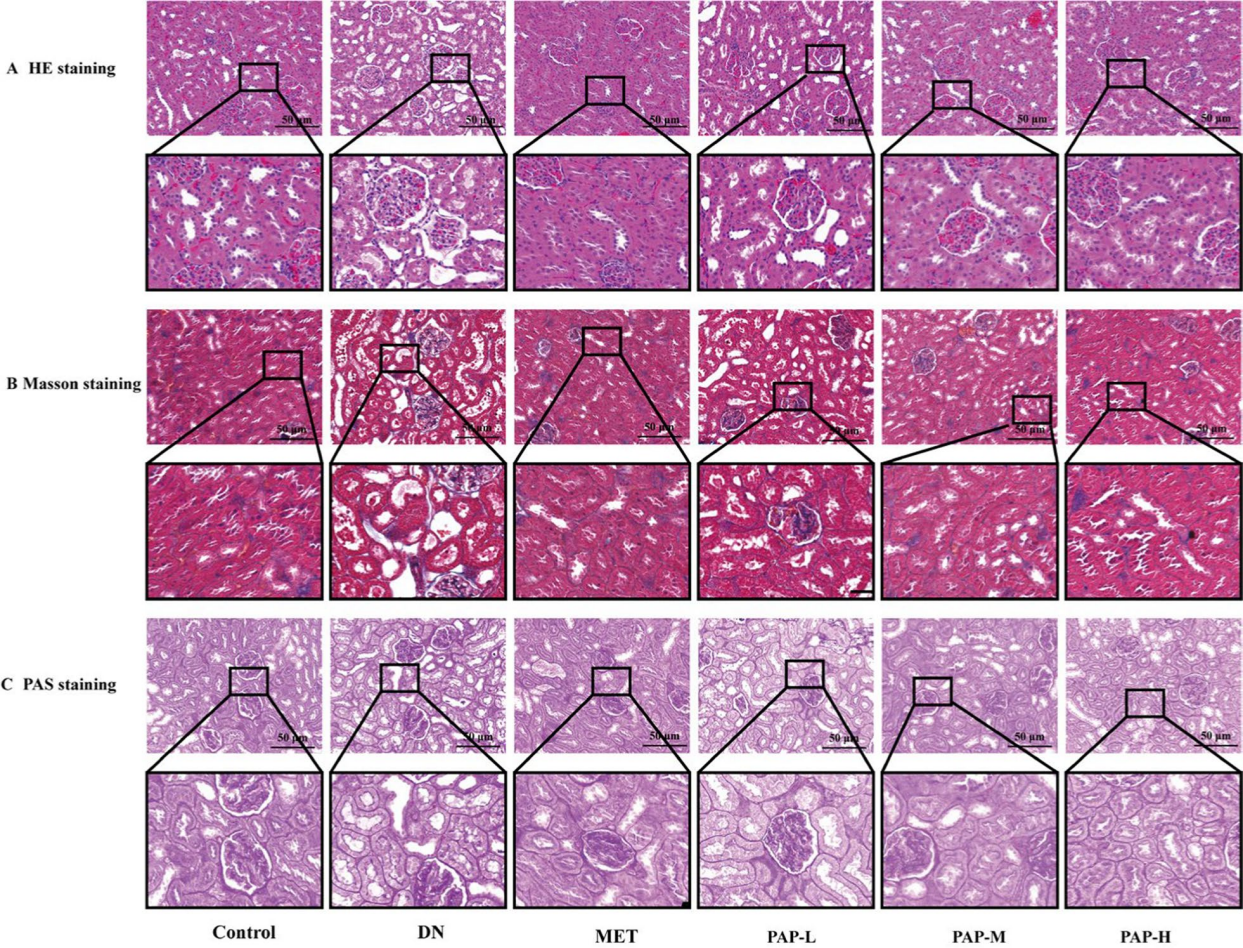
Effects of PAP treatment on colon and renal histopathology changes. **A** Representative histological images of renal HE staining. **B** Representative histological images of renal Masson staining. **C** Representative histological images of renal PAS staining

The results of HE staining, Masson staining and PAS staining are shown in Fig. 3. HE staining demonstrated intact glomerular architecture with unremarkable mesangial matrix and preserved tubular structures in the control group. In contrast, the DN group exhibited significant renal necrosis, glomerular atrophy, and extensive tubular epithelial degeneration accompanied by inflammatory infiltration. The PAP-treated group showed marked improvements in glomerular basement membrane thickening and mesangial matrix proliferation. Masson staining revealed minimal collagen deposition in glomerular basement membranes and tubulointerstitial areas of control specimens. Pathological analysis identified substantial blue-stained collagen fiber accumulation in both glomeruli and renal tubules of model animals. Notably, PAP-treated rats demonstrated significantly reduced collagen fiber deposition compared to the DN group. PAS staining revealed significantly enhanced PAS-positive expression in the glomerular mesangial areas of the DN group compared to the control group, whereas the PAP-treated group exhibited a marked reduction in positive staining intensity relative to the DN group. These findings indicate substantial mitigation of reducing sugar accumulation in renal tissues through PAP administration, demonstrating its potent capacity to downregulate reducing sugar levels in the nephric parenchyma.

### Effects of PAP on oxidative stress, inflammatory response, fibrosis, and apoptosis signaling pathway in DN rats

#### PAP activated the renal PI3K/AKT signaling pathway in DN rats

**Fig. 4:**
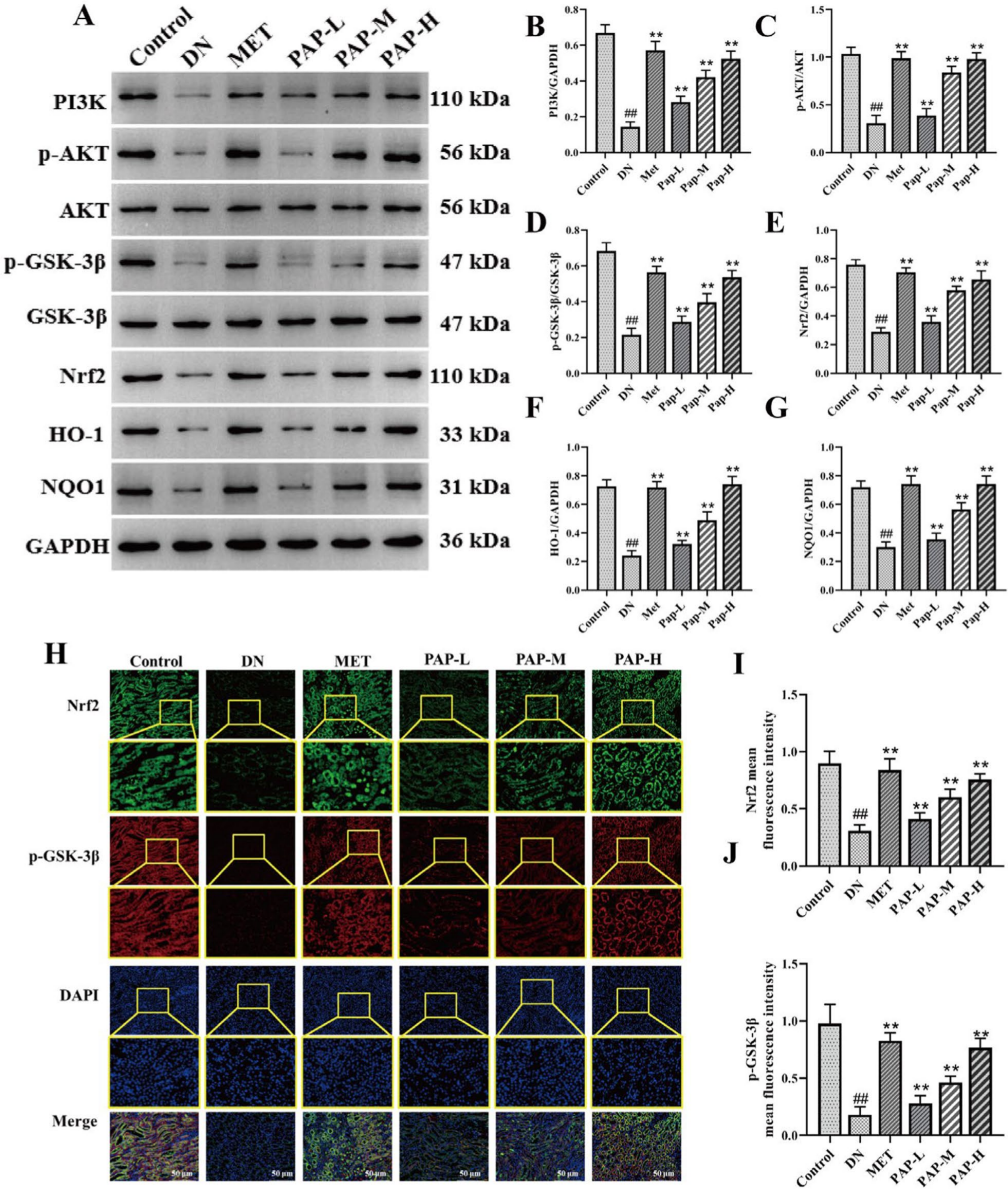
The effect of PAP on the PI3K/AKT pathway in DN rats (n = 3). **A** The protein expression of PI3K, p-AKT, AKT, p-GSK-3β, GSK-3β, Nrf2, HO-1, NQO1 in kidney. **B** The protein expression of PI3K/GAPDH. **C** The protein expression of p-AKT/PI3K. **D** The protein expression of p-GSK-3β/GSK-3β. **E** The protein expression of Nrf2/GAPDH. **F** The protein expression of HO-1/ GAPDH. **G** The protein expression of NQO1/GAPDH. **H** Immunofluorescence detection of Nrf2 and p-GSK-β expression, 200×, scale bar = 50 μm. **I** Optical density analysis of Nrf2. **J** Optical density analysis of p-GSK-β.). ^##^*p* < 0.01 vs. Control group; *p < 0.05, **p < 0.01 vs. the DN group

PI3K/Akt signaling pathway is involved in a variety of cellular activities, mediating glucose homeostasis, lipid metabolism, autophagy and apoptosis, inflammation, oxidative stress and other key physiological processes, and plays an important role in the pathogenesis of DN [32]. The PI3K/AKT signaling pathway-related proteins were determined by Western blotting in rats (Fig. 4). Compared with the Control group, the levels of PI3K, p-AKT, AKT, p-GSK-3β, GSK-3β, Nrf2, HO-1 and NQO1 in the DN group were significantly decreased, while PAP significantly reversed these effects (Fig. 4B, E–G). In addition, compared with the Control group, the levels of p-AKT/AKT and p-GSK-3β/GSK-3β in the DN group were significantly decreased, while PAP significantly reversed these effects (Fig. 4C, D). The immunofluorescence results simultaneously showed that the expressions of Nrf2 and p-GSK-β in renal tissue of the DN group were significantly decreased, further indicating that DN induces oxidative stress injury in renal tissue (Fig. 4H–J). After PAP intervention, the PI3K/AKT signaling pathway was activated, the expression of related proteins was significantly up-regulated (*p* < 0.01), and the antioxidant activity was improved.

#### PAP inhibits kidney TGF-β/Smad signaling pathway in DN rats

**Fig. 5:**
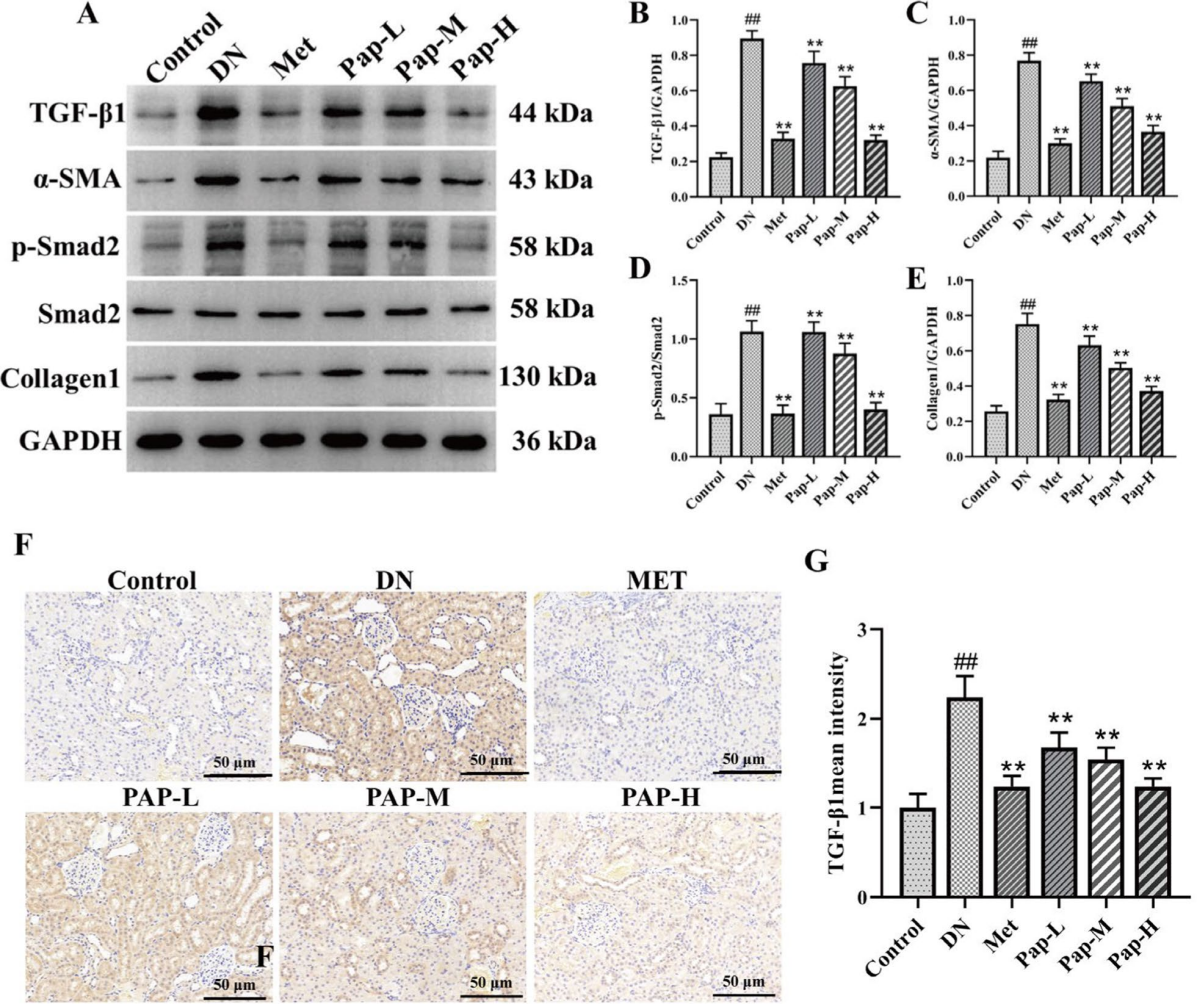
The effect of PAP on the TGF-β/Smad pathway in the kidney (n = 3). **A** The protein expression of TGF-β, α-SMA, p-Smad2, Smad2, GSK-3β, Collagen1 in kidney. **B** The protein expression of TGF-β/GAPDH. **C** The protein expression of α-SMA/GAPDH. **D** The protein expression of p-Smad2/GAPDH. **E** The protein expression of Collagen1/GAPDH. **F** Immunofluorescence detection of TGF-β expression, 200×, scale bar = 50 μm. **G** Optical density analysis of TGF-β). ^##^*p* < 0.01 vs. Control group; *p < 0.05, **p < 0.01 vs. the DN group

To elucidate the underlying mechanism of PAP on renal fibrosis in DN rats, the TGF-β/Smad signaling pathway was assessed by Western blot (Fig. 5). Compared with the control group, the levels of TGF-β, α-SMA, GSK-3β, and Collagen1 were significantly increased in the DN group, while PAP significantly reversed these effects (Fig. 5B, D, E). In addition, compared with the control group, the p-Smad2/Smad2 level was significantly increased in the DN group, while PAP significantly decreased the expression of p-Smad2/Smad2 (Fig. 5C). Meanwhile, immunofluorescence results showed that the expression of Nrf2 and p-GSK-β in renal tissues was significantly reduced in the PAP group, further indicating that PAP could alleviate renal tissue fibrosis in DN (Fig. 5F, G). The results demonstrated that PAP intervention treatment blocked the TGF-β/Smad pathway, significantly inhibited the expression of related proteins (*p* < 0.01), and alleviated renal fibrosis.

#### PAP inhibits the renal apoptosis signaling pathway in DN rats

**Fig. 6:**
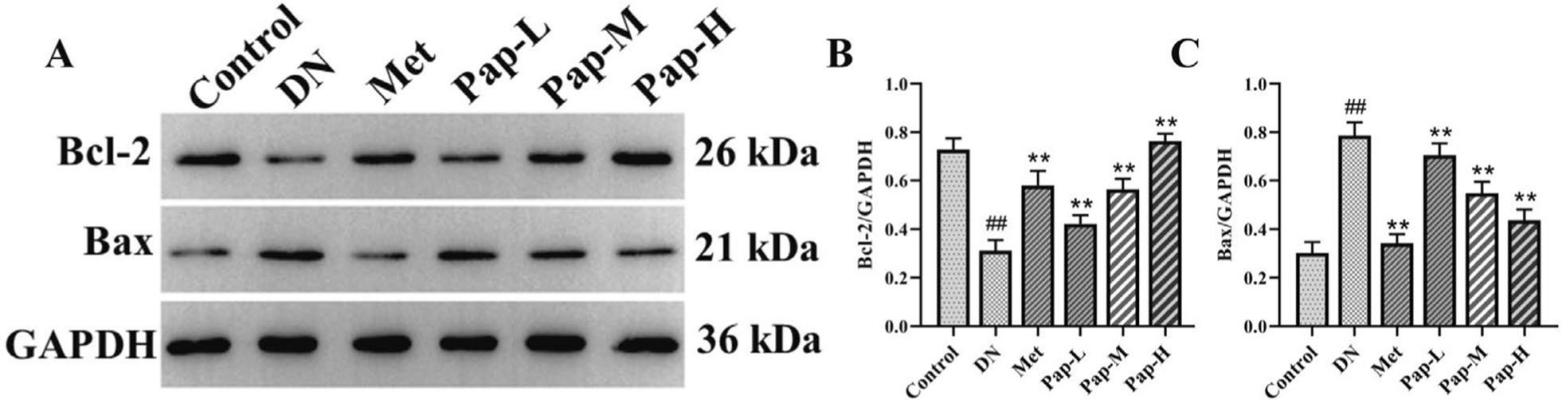
The effect of PAP on the Apoptosis pathway in the kidney (n = 3). **A** The protein expression of Bcl-2, Bax. **B** The protein expression of Bcl-2. **C** The protein expression of Bax. ^##^*p* < 0.01 vs. Control group; *p < 0.05, **p < 0.01 vs. the DN group

To elucidate the potential mechanism of PAP on apoptosis in DN rats, the apoptotic signaling pathway was determined by Western blotting (Fig. 6). Compared with the control group, the level of Bcl-2 in the DN group was significantly decreased, and the level of Bax was significantly increased, while PAP significantly improved the expression of p-Bcl-2 and Bax (Fig. 6B, C). The results showed that PAP could significantly inhibit the protein expression of apoptosis signaling pathway in DN rats.

### PAP modulates intestinal flora in DN rats

**Fig. 7:**
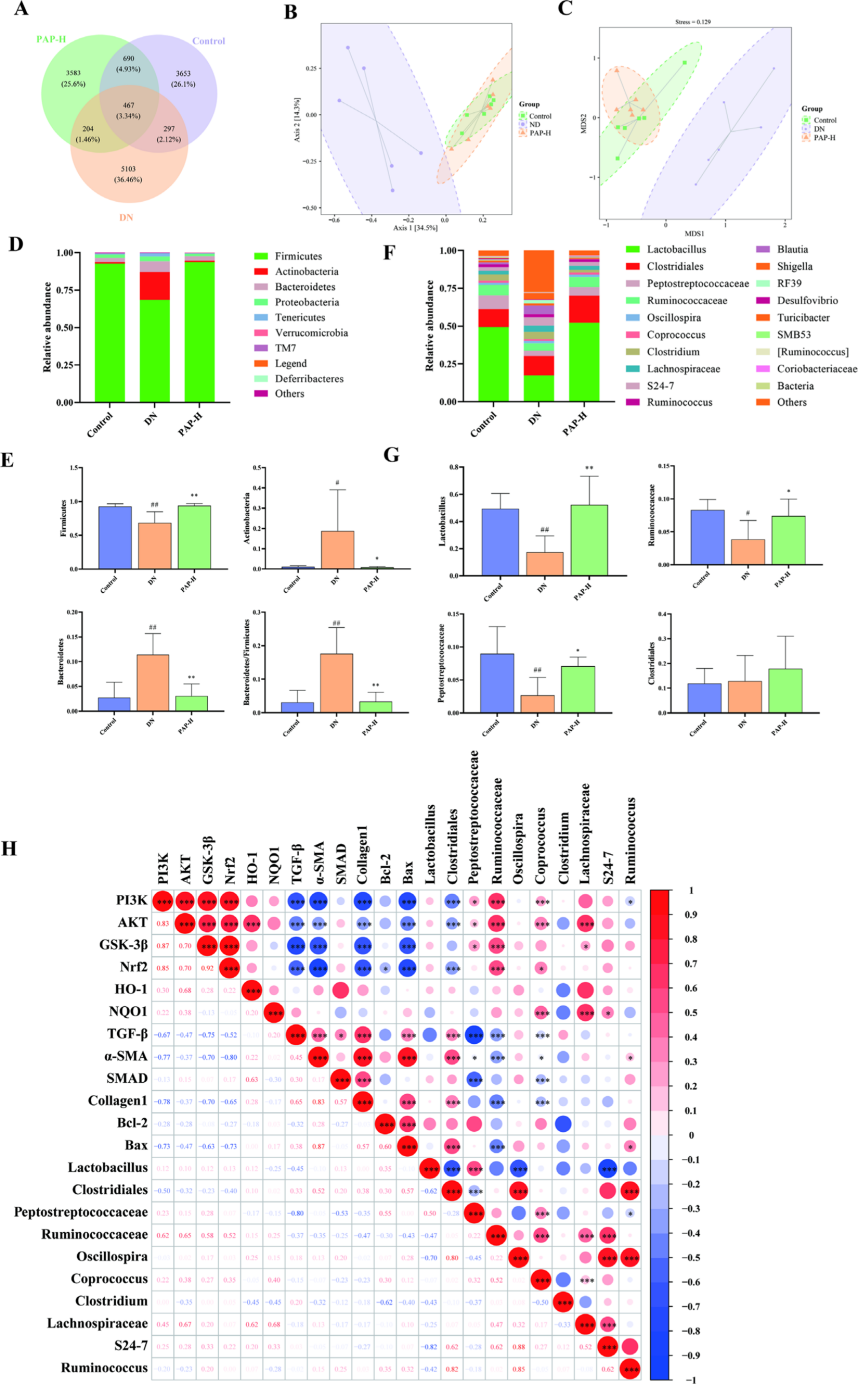
PAP modulates intestinal flora in DN rats. **A** The result of OTU. **B** The result of PCoA. **C** The result of NMDS. **D** Bacterial community structure at phylum level. **E** Bacterial community structure of the top 4 at phylum level. **F** Bacterial community structure at genus level. **G** Bacterial community structure of the top 4 at genus level. **H** genus level difference between flora and pathway index correlation analysis). ^##^*p* < 0.01 vs. Control group; *p < 0.05, **p < 0.01 vs. the DN group

The paired-end sequencing of community DNA fragments was performed on the Illumina platform. These sequences underwent primer removal, quality filtering, denoising, merging, and chimera elimination using the DADA2 algorithm. All groups demonstrated sequencing effectiveness with valid sequences per sample exceeding 4 × 10^4^, indicating high-quality sequencing data suitable for subsequent analyses. OTUs were clustered at 97% similarity threshold through QIIME 2 software, yielding 4944 OTUs in the Control group, 5107 OTUs in the DN group, and 6071 OTUs in the PAP-H group (Fig. 7A).

PCoA based on the Bray–Curtis distance algorithm was plotted (Fig. 7B), and NMDS analysis using the Jaccard distance algorithm was performed (Fig. 7C). The results revealed relatively minor differences within each group. The DN group exhibited significant separation from the remaining two groups, while the Control and PAP-H groups showed partial overlap, indicating that PAP could modulate structural changes in the gut microbiota of rats.

The phylum-level analysis of samples (Fig. 7D, E) identified *Firmicutes*, *Actinobacteria*, and *Bacteroidetes* as dominant phyla, with *Firmicutes* and *Actinobacteria* being predominant. Intergroup comparisons revealed that compared to the Control group, the DN group exhibited significant or highly significant reductions in *Firmicutes* abundance, accompanied by significant or highly significant increases in *Actinobacteria*, *Bacteroidetes*, and B/F ratio. Following PAP-H intervention, *Firmicutes* demonstrated a highly significant increase, while *Actinobacteria*, *Bacteroidetes*, and *Bacteroidetes*/ *Firmicutes* (B/F) ratio showed significant or highly significant decreases.

The analysis at the genus level (Fig. 7F, G) revealed *Lactobacillus, Clostridiales*, *Peptostreptococcaceae*, and *Ruminococcaceae* as the dominant genera. Comparison of richness variations between groups demonstrated that compared to the Control group, Lactobacillus, *Peptostreptococcaceae*, and *Ruminococcaceae* were significantly or extremely significantly reduced in the DN group, while *Clostridiales* showed no significant change. Following PAP-H administration, significant or extremely significant increases were observed in Lactobacillus, *Peptostreptococcaceae*, and *Ruminococcaceae*. Interestingly, PAP-H elevated *Clostridiales* in rats, whereas no significant change of *Clostridiales* was detected in DN rats.

To explore the relationship between gut microbiota and protein expression, we conducted a correlation analysis on the relative abundances of the top 10 differential bacterial genera and key proteins in DN rats. The strength of these correlations was determined using the correlation coefficient (r), where |r|> 0.7 indicates a strong correlation, 0.4 <|r|≤ 0.7 a moderate correlation, and 0.2 <|r|≤ 0.4 a weak correlation (Fig. 7H). The analysis revealed that *Lactobacillus*, *Oscillospira*, and *Clostridium* did not exhibit any significant correlation with the measured proteins (*p* > 0.05). However, several significant correlations were identified: *Clostridiales* was negatively correlated with PI3K, AKT, and Nrf2, while being positively correlated with TGF-β, α-SMA, Collagen1, and Bax. P*eptostreptococcaceae* showed positive correlations with PI3K, AKT, and GSK-3β, and negative correlations with TGF-β and α-SMA. *Ruminococcaceae* was positively correlated with PI3K, AKT, GSK-3β, and Nrf2, but negatively correlated with TGF-β, α-SMA, Collagen1, and Bax. *Coprococcus* displayed positive correlations with PI3K, AKT, and Nrf2, and negative correlations with TGF-β, α-SMA, SMAD, and Collagen1. Furthermore, *Lachnospiraceae* was positively correlated with AKT and GSK-3β, S24-7 with NQO1, and *Ruminococcus* was negatively correlated with PI3K but positively correlated with Bax. In addition, we observed significant inter-correlations among the signaling pathways themselves. Proteins of the PI3K/AKT pathway were negatively correlated with those of both the TGF-β/Smad and apoptosis pathways. Conversely, a positive correlation was found between proteins of the TGF-β/Smad and apoptosis pathways. These results suggest a significant correlation between the gut microbiota and protein markers relevant to PAP therapeutic effect on DN. Moreover, they highlight the interplay among the PI3K/AKT, TGF-β/Smad, and apoptosis signaling pathways.

### Effects of PAP on oxidative stress, inflammatory response, fibrosis, and apoptosis signaling pathway in HK-2 cells

#### PAP protected HG-induced HK-2 cells against PI3K/AKT

**Fig. 8:**
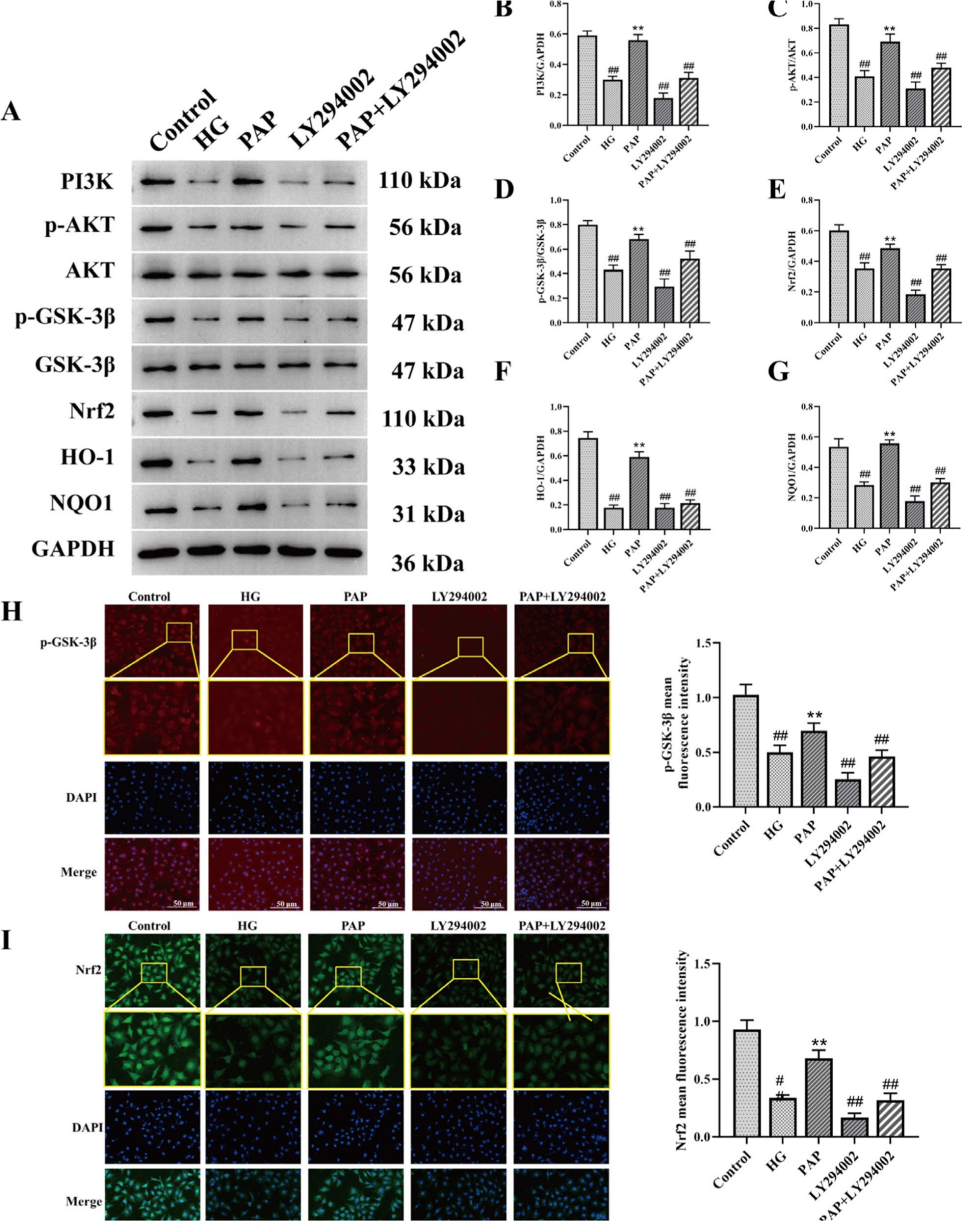
The effect of PAP on the PI3K/AKT pathway in HK-2 cell. **A** The protein expression of PI3K, p-AKT, AKT, p-GSK-3β, GSK-3β, Nrf2, HO-1, NQO1. **B** The protein expression of PI3K/GAPDH. **C** The protein expression of p-AKT/PI3K. **D** The protein expression of p-GSK-3β/GSK-3β. **E** The protein expression of Nrf2/GAPDH. **F** The protein expression of HO-1/GAPDH. **G** The protein expression of NQO1/GAPDH. **H** Immunofluorescence detection of p-GSK-3β expression, 200×, scale bar = 50 μm. **I** Immunofluorescence detection of Nrf222 expression, 200×, scale bar = 50 μm. ^##^*p* < 0.01 vs. Control group; *p < 0.05, **p < 0.01 vs. the HG group

The protein levels of PI3K/AKT signaling pathway-related components in HK-2 cells were determined by Western blot analysis (Fig. 8). Compared with the Control group, the HG group exhibited significantly decreased levels of PI3K, p-AKT, AKT, p-GSK-3β, GSK-3β, Nrf2, HO-1, and NQO1, which were significantly reversed by PAP treatment. LY294002 stimulation profoundly reduced the levels of PI3K, p-AKT, AKT, p-GSK-3β, GSK-3β, Nrf2, HO-1, and NQO1, while PAP administration significantly ameliorated antioxidant capacity in HK-2 cells (Fig. 8B, E–G). Furthermore, the p-AKT/AKT and p-GSK-3β/GSK-3β ratios were significantly reduced in the Model group compared with the Control group, and these changes were substantially reversed by PAP intervention (Fig. 8C, D). Immunofluorescence results further demonstrated significantly decreased p-GSK-3β and Nrf2 expression in renal tissues of the HG group, indicating oxidative stress injury mediated by DN. These findings suggest that PAP intervention activates the PI3K/AKT pathway, significantly upregulates the expression of associated proteins, and enhances antioxidant activity.

#### PAP protected HG-induced HK-2 cells against fibrosis

**Fig. 9:**
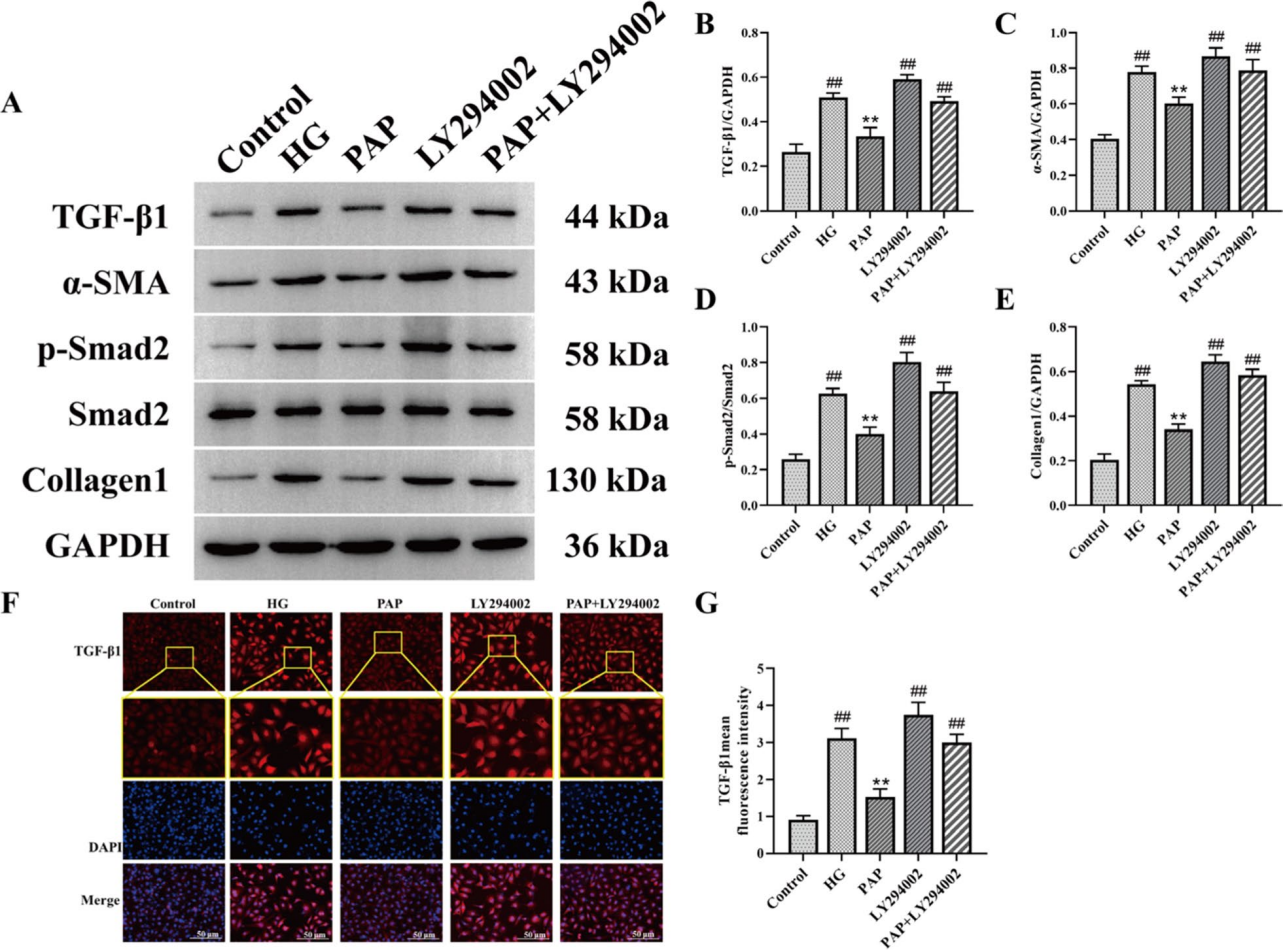
PAP protected HG-induced HK-2 cells against fibrosis. **A** The protein expression of TGF-β, α-SMA, p-Smad2, Smad2, Collagen1 in HK-2 cell. **B** The protein expression of TGF-β/GAPDH. **C** The protein expression of α-SMA/ GAPDH. **D** The protein expression of p-Smad2/ Smad2. **E** The protein expression of Collagen1/ GAPDH. **F** Immunofluorescence detection of TGF-βexpression, 200×, scale bar = 50 μm. **G** fluorescence intensity of TGF-β). ^##^*p* < 0.01 vs. Control group; *p < 0.05, **p < 0.01 vs. the HG group

The protein levels of TGF-β/Smad signaling pathway-related proteins in HK-2 cells were measured by Western blot (Fig. 9). Compared to the Control group, the levels of TGF-β, α-SMA, p-Smad2/Smad2, and Collagen1 were significantly increased in the HG group, whereas PAP markedly reduced the expression of these proteins. After LY294002 stimulation, the levels of TGF-β, α-SMA, p-Smad2/Smad2, and Collagen1 were again significantly elevated, but these increases were alleviated by PAP treatment in HK-2 cells (Fig. 9B–E). Immunofluorescence results further demonstrated that TGF-β expression in the PAP group was significantly lower than in the HG group, indicating that PAP attenuated high glucose-induced cellular fibrosis (Fig. 9F, G). These results suggest that PAP intervention blocks the TGF-β/Smad pathway, significantly inhibits the expression of related proteins, and alleviates cellular fibrosis.

#### PAP protected HG-induced HK-2 cells against apoptosis

**Fig. 10:**
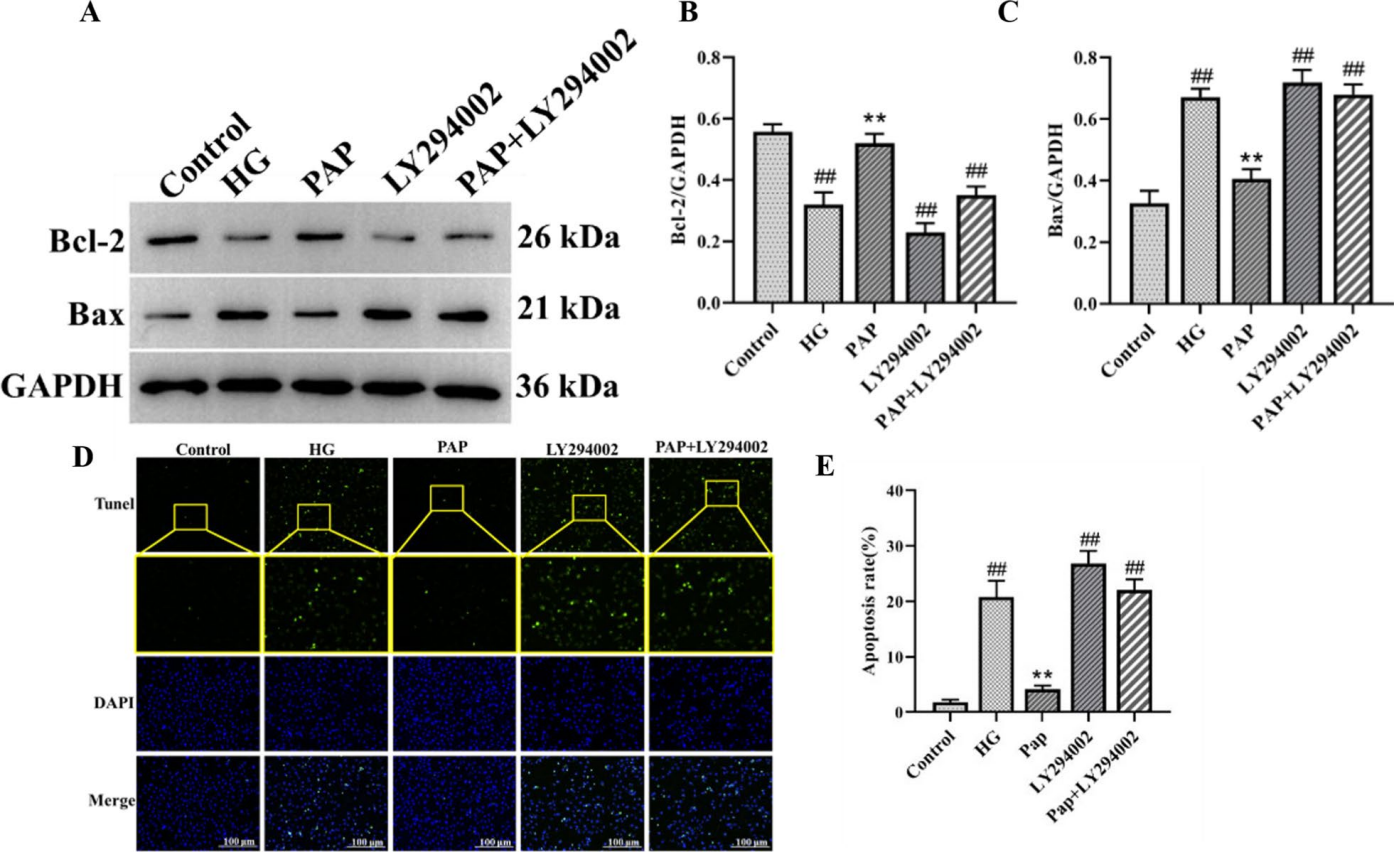
The effect of PAP on the Apoptosis pathway in HK-2 cell. **A** The protein expression of Bcl-2, Bax. **B** The protein expression of Bcl-2. **C** The protein expression of Bax. **D** The result of Tunel staining. **E** The result of apoptosis rates. ^##^*p* < 0.01 vs. Control group; *p < 0.05, **p < 0.01 vs. the HG group

The apoptosis signaling pathway was assessed by Western blot analysis (Fig. 10). Compared with the Control group, the HG group exhibited a significant decrease in Bcl-2 levels and a marked increase in Bax levels. PAP treatment significantly ameliorated the expression of p-Bcl-2 and Bax (Fig. 10B, C). Tunel staining results further demonstrated that apoptosis expression in the PAP group was significantly lower than in the HG group, indicating that PAP attenuated high glucose-induced cellular fibrosis (Fig. 10D, E). These results demonstrate that PAP effectively suppressed the protein expression in the apoptosis signaling pathway of HK-2 cells.

## Discussion

Diabetic nephropathy (DN), as the most prevalent microvascular complication of diabetes, with a significant impact on global human health quality. However, current therapeutic approaches remain unsatisfactory. Despite the established anti-diabetic properties of PAP, the underlying mechanisms remain incompletely understood. This study investigates the role of PAP in DN through both animal experiments and cellular studies, aiming to provide novel therapeutic directions for DN management.

Urinary protein, blood glucose, renal function parameters, and histopathology have been identified as the primary clinical features of DN [43]. Scr and BUN are specific indicators for clinically evaluating renal function. In the event of renal injury occurs or glomerular filtration capacity declines, the filtration of BUN and Scr is reduced, leading to elevated levels of BUN and Scr in the blood. Abnormal glucose metabolism has been demonstrated to induce alterations in renal hemodynamics and to increase FBG levels [3]. Oxidative stress has been demonstrated to exacerbate collagen fiber production in renal tissues and glomerulosclerosis, impairing glomerular barrier function and persistently aggravating proteinuria [29]. SOD is a key antioxidant enzyme that regulates oxidative stress during renal injury. Malondialdehyde MDA, as the terminal product of lipid peroxidation, serves as a biomarker reflecting the degree of oxidative damage. GSH as been shown to catalyse the reduction of hydrogen peroxide to water or the corresponding alcohols, thus counteracting oxidative stress. In this study, PAP significantly reduced serum levels of FBG, 24-h urinary protein, Scr, BUN, and renal MDA content in DN rats, while increasing renal SOD and GSH levels. Furthermore, HE staining, Masson staining, and PAS staining results demonstrated that PAP effectively ameliorated renal histopathological lesions in DN rats.

Oxidative stress has been demonstrated to play a critical role in the pathogenesis of diabetic nephropathy. has been demonstrated to activate various signalling pathways, including PKC, MAPK, JAK-STAT, and the hexosamine pathway, ultimately leading to increased glomerular extracellular matrix, cellular senescence, apoptosis, inflammation, tubulointerstitial fibrosis, and progression to end-stage renal disease [13, 21]. The PI3K/AKT pathway regulates diverse biological processes, including cell proliferation, differentiation, and apoptosis, under both physiological and pathological conditions [31]. Nrf2 activation is regulated by PI3K, with current studies indicating PI3K involvement in Nrf2 activation [2] PI3K modulates actin arrangement and regulates actin depolymerization, leading to the dissociation of Nrf2 and Keap1.. As a pivotal downstream kinase in the PI3K signalling pathway, AKT phosphorylation serves as a reflection of PI3K activation status. In this study, decreased expression of p-AKT and Nrf2 proteins was observed with disease progression, suggesting that during early-stage diabetic nephropathy, the body consumes endogenous Nrf2 signaling to enhance antioxidant protein expression, thereby boosting antioxidant capacity and mitigating oxidative damage.

ROS can activate TGF-β [9]. As a pivotal cytokine in diabetic nephropathy pathogenesis, TGF-β mediates apoptosis and transdifferentiating of renal tubular epithelial cells via the TGF-β-Smad2/3 signaling pathway, thereby promoting extracellular matrix (ECM) formation in the kidney and subsequent progression to DN-related renal fibrosis [18]. TGF-β1 can activate PI3K through a non-Smad pathway, thereby phosphorylating Akt and amplifying its pro-fibrotic and epithelial-mesenchymal transition (EMT) effects [45]. Activation of the TGF-β/Smad signaling pathway enhances ECM production and increases α-SMA expression in cells, leading to excessive ECM deposition, structural damage to the kidney, and loss of renal function [35]. The PI3K/AKT signaling pathway and TGF-β/Smad signaling pathway jointly regulate key nodes such as mTOR and GSK-3β. Upon activation by Akt, mTOR not only promotes ECM synthesis but also stabilizes the Smad complex and enhances its transcriptional efficiency. By enhancing antioxidant capacity, PAP inhibits high glucose-induced oxidative stress injury, attenuates renal fibrosis, and delays the pathological progression of DN.

GSK-3β, as a downstream protein of AKT, extensively participates in the regulation of mitochondrial function [40]. AKT is activated through phosphorylation, and the activated AKT (p-AKT) regulates GSK-3β [16]. Subsequently, activated GSK-3β modulates the Bax/Bcl-2 ratio, thereby influencing mitochondrial membrane permeability. This stimulates the opening of the mitochondrial permeability transition pore, promotes CytoC release from mitochondria, and ultimately contributes to the regulation of apoptosis. This study found that LY294002 significantly suppressed AKT phosphorylation, indirectly downregulated TGF-β1 expression and Smad2 phosphorylation levels, thereby reducing ECM accumulation and renal tubular injury. This demonstrates that PI3K/AKT is one of the key upstream regulators of the TGF-β/Smad pathway, consistent with previous literature reports [39].

The correlation between gut microbiota α-diversity and (DN remains controversial due to inconsistent findings across studies. Some research has demonstrated no significant association between α-diversity of gut microbiota and DN [44], which is corroborated by our experimental results. *Firmicutes* and *Bacteroidetes* are the two most prominent phyla linked to energy metabolism homeostasis in the gastrointestinal tract. Butyrate, a classic metabolite of *Firmicutes*, plays a crucial modulatory role during inflammatory responses [8, 37]. Studies report an increasing trend in the B/F ratio in the gut microbiota of DN patients and rodent DN models [44]. At the genus level, PAP increased intestinal abundances of Lactobacillus, *Ruminococcaceae*, and *Clostridiales* in rats. Reduced Lactobacillus abundance has been observed in models of diabetes, fatty liver disease, and obesity [23], while Lactobacillus intake in DN patients significantly improves glycemic control [15]. *Ruminococcaceae* typically responds to Chlorella pyrenoidosa treatment in rats, suggesting its potential role in diabetes management [34]. *Clostridiales*, belonging to Firmicutes, inhibits pathogens via antimicrobial secretion. Although limited studies report its role in DN, this experiment found no significant difference in *Clostridiales* abundance between DN models and controls, whereas PAP markedly increased its abundance (*p* > 0.05). Gut microbiota analysis indicates that PAP alleviates DN symptoms by reducing the B/F ratio, suppressing renal inflammation, and modulating blood glucose levels.

Gut microbiota-derived metabolites mediate and drive interactions between cells, organs, and the host with the environment. A growing body of evidence indicates that metabolic dysfunction associated with the gut microbiota accelerates oxidative stress, inflammatory responses, and apoptosis in DN rats [4, 41, 42]. Here, we demonstrate that PAP can directly or indirectly control gut microbiota during both the progression and treatment of DN, thereby alleviating oxidative stress, inflammatory responses, and apoptosis in DN rats. *Peptostreptococcaceae* is positively correlated with the PI3K/AKT signaling pathway and negatively correlated with the TGF-β/Smad signaling pathway and apoptosis signaling pathway [10, 36, 38], which is consistent with our data. *Coprococcus* is a butyrate-producing bacterium, and butyrate plays a crucial role in colonic cell metabolism and gut health, potentially related to gut permeability. Increased gut permeability can promote the translocation of bacteria and their products, leading to chronic inflammation [28]. We found that *Coprococcus* is not only positively correlated with inflammation but also negatively correlated with oxidative stress. In summary, our study investigated the relationship between gut microbiota and the protein expression of oxidative stress, inflammation, and apoptosis signaling pathways in DN rats. Concurrently, we also found strong correlations between the PI3K/AKT signaling pathway and TGF-β and apoptosis.

## Conclusion

This study reveals that PAP ameliorates DN symptoms by enhancing antioxidant capacity, alleviating renal fibrosis, and reducing apoptosis via gut microbiota-related pathways. PAP could serve as a promising candidate natural product for DN amelioration.

## Data Availability

The data supporting the results of this study can be obtained from the corresponding author on reasonable request.
